# Meta-analysis of gene expression profiles in long-term non-progressors infected with HIV-1

**DOI:** 10.1186/s12920-018-0443-x

**Published:** 2019-01-09

**Authors:** Sun Young Lee, Yong Kwang Park, Cheol-Hee Yoon, Kisoon Kim, Kyung-Chang Kim

**Affiliations:** Division of Viral Disease Research, Center for Infectious Disease Research, Korea National Institute of Health, 187 Osongsaengmyeong 2-ro, Cheongju, Chungbuk 28159 Republic of Korea

**Keywords:** HIV-1, Long-term non-progressors, Gene expression profile, Biomarkers, Meta-analysis

## Abstract

**Background:**

In the absence of antiretroviral treatments (ARTs), a small group of individuals infected with HIV, including long-term non-progressors (LTNPs) who maintain high levels of CD4+ T cells for more than 7–10 years in the absence of ART and in particular a subgroup of LTNPs, elite controllers (ECs), who have low levels of viremia, remain clinically and/or immunologically stable for years. However, the mechanism of stable disease progression in LTNPs and ECs needs to be elucidated to help those infected with HIV-1 remain healthy. In this study, to identify the characteristics of gene expression profiles and biomarkers in LTNPs, we performed a meta-analysis using multiple gene expression profiles among LTNPs, individuals infected with HIV-1 without ART, individuals infected with HIV-1 with ART, and healthy controls.

**Methods:**

The gene expression profiles obtained from the Gene Expression Omnibus (GEO) microarray data repositories were classified into three groups: LTNPs versus healthy controls (first group, 3 studies), LTNPs versus patients infected with HIV-1 without ART (second group, 3 studies), and LTNPs versus patients infected with HIV-1 with ART (third group, 3 studies). In addition, we considered a fourth group, patients infected with HIV-1 without ART versus healthy controls (3 studies), to exclude genes associated with HIV-1 infection in the three groups. For each group, we performed a meta-analysis using the *RankProd* method to identify and compare the differentially expressed genes (DEGs) in the three groups.

**Results:**

We identified the 14 common DEGs in the three groups when comparing them with each other. Most belonged to immune responses, antigen processing and presentation, the interferon-gamma-mediated signaling pathway, and T cell co-stimulation. Of these DEGs, *PHLDA1* was up-regulated and *ACTB* and *ACTG1* were down-regulated in all three groups. However, the rest of the up- or down-regulated genes were discordant in the three groups. Additionally, *ACTB* and *ACTG1* are known to inhibit viral assembly and production, and *THBS1* is known to inhibit HIV-1 infection.

**Conclusions:**

These results suggest that significant genes identified in a meta-analysis provide clues to the cause of delayed disease progression and give a deeper understanding of HIV pathogenesis in LTNPs.

**Electronic supplementary material:**

The online version of this article (10.1186/s12920-018-0443-x) contains supplementary material, which is available to authorized users.

## Background

In the absence of antiretroviral treatments (ARTs), most individuals infected with HIV exhibit progressive active viral transcription and replication, which result into the loss of CD4+ T cells, appearance of clinical symptoms, and disease progression. In contrast, a small group of individuals infected with HIV, including long-term non-progressors (LTNPs) who maintain high levels of CD4+ T cells for more than 7–10 years in the absence of ART and especially a subgroup of LTNPs, elite controllers (ECs), who have low levels of viremia, remain clinically and/or immunologically stable for years. Understanding the mechanisms of delayed disease progression in LTNPs would be valuable for HIV disease management and treatment, but they remain poorly understood. Previous studies using gene expression profiling [1–5] and meta-analysis [6] in LTNPs have identified that the genes related to interferon responses are a signature for progression. It is usually not advisable to directly combine or compare the gene expression values from different gene expression data sets because of their inherent heterogeneity (i.e., different platforms, protocols, and so on). Instead, the integration of various expression data sets is performed on the summary-level data such as *P* values, effect sizes, or gene ranks. Meta-analysis, a statistical approach that combines results from independent but related studies, has the potential to increase both the statistical power and generalizability of single-study analysis [7] and is most commonly used for the purpose of detecting differentially expressed genes (DEGs) [8]. The application of meta-analysis to clinical datasets has demonstrated that it achieves more reliable identification than an individual analysis and more robust rank products in gene ranking, which lead to much higher reproducibility among independent studies [9]. Thus, to identify the characteristics of gene expression profiles and biomarkers or factors in LTNPs or understand the factors specifically associated with LTNPs, we performed a meta-analysis of gene expression profiles in LTNPs. The gene expression profiles obtained from the Gene Expression Omnibus (GEO) microarray data repositories [10] were classified into four groups. For each group, we performed the meta-analysis using the *RankProd* [11] method to identify DEGs in LTNPs and then compared the DEGs of each group. In this study, we performed a meta-analysis of gene expression profiles in LTNPs to identify the characteristics of delayed disease progression and provide a deeper understanding of HIV pathogenesis.

## Methods

### Procedure

**Fig. 1:**
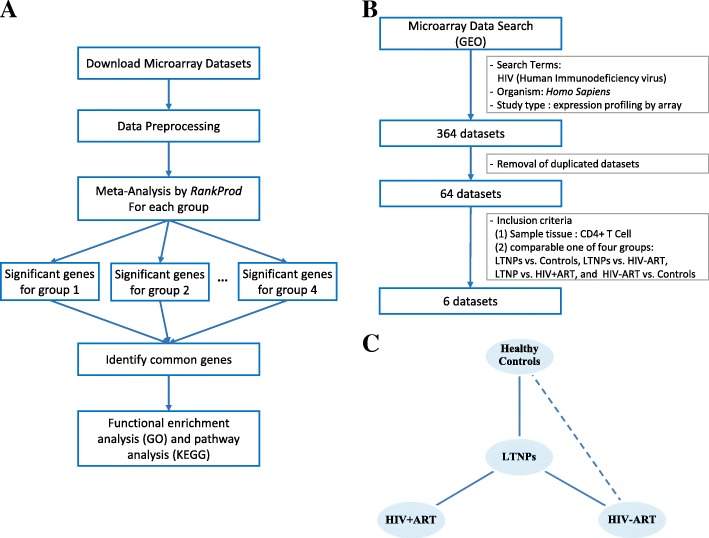
Study workflow. **a** The process used for meta-analysis. **b** Identification of eligible gene expression datasets for meta-analysis. **c** Pair-wise groups for meta-analysis. LTNPs, long-term non-progressors; HIV-ART, individuals infected with HIV-1 without ART; HIV + ART, individuals infected with HIV-1 with ART

We performed the analysis in accordance with the workflow illustrated in Fig. 1a for the meta-analysis of gene expression profiles in LTNPs and to identify candidate genes that characterized LTNPs. First, we obtained the microarray datasets from GEO microarray data repositories. After removing duplicates and irrelevant datasets, six microarray datasets were considered and classified into four groups according to the inclusion criteria. Next, we identified the significant genes in each group using the meta-analysis method *RankProd* [11] and selected potential biomarkers for LTNPs from these significant genes. Finally, Functional enrichment analysis (Gene ontology (GO)) and pathway analysis (Kyoto Encyclopedia of Genes and Genomes, KEGG) were performed.

### Microarray datasets

As shown Fig. 1b, we searched the gene expression profiles using the keywords “Human Immunodeficiency virus/HIV” in GEO microarray data repositories. The datasets were selected using the following criteria: tissue section with peripheral blood mononuclear cell (PBMC); gene expression data; and the species *Homo sapiens*. After removing duplicates and irrelevant datasets, a total of 64 microarray datasets were considered. Of these, six datasets were finally selected in accordance with the inclusion criteria. The inclusion criteria were as follows: tissue with CD4+ T cell and mutual comparability in four groups (Fig. 1c), including LTNPs versus healthy controls (LTNPs vs. Controls), LTNPs versus patients infected with HIV-1 without ART (LTNPs vs. HIV-ART), LTNPs versus patients infected with HIV-1 with ART (LTNPs vs. HIV + ART), and patients infected with HIV-1 without ART versus healthy controls (HIV-ART vs. Controls). Here, patients infected with HIV-1 without ART were chronic patients 1 year after HIV infection. Six datasets were organized into four groups (Table 1): LTNPs versus healthy controls (first group, 3 studies, LC), LTNPs versus patients infected with HIV-1 without ART (second group, 3 studies, LH), LTNPs versus patients infected with HIV-1 with ART (third group, 3 studies, LA), and patients infected with HIV-1 without ART versus healthy controls (fourth group, 3 studies, HC).

**Table 1:** Summary of datasets analyzed in the meta-analysis

Series Dataset GEO ID		No. of Samples		Sample Source	Platform
		Case	Control		
		LTNPs	Controls		
1 (LC)	GSE6740 [1]	5	5	CD4+ T cell	GPL96, Affymetrix Human Genome U133A array
	GSE18233 [51]	16	8	CD4+ T cell	GPL6884, Illumina Human WG-6 v3.0 expression bead chip
	GSE23879 [2]	12	12	CD4+ T cell	GPL8432, Illumina HumanRef-8 WG-DASL V3.0
		LTNPs	HIV-ART		
2 (LH)	GSE6740 [1]	5	5	CD4+ T cell	GPL96, Affymetrix Human Genome U133A array
	GSE18233 [51]	16	73	CD4+ T cell	GPL6884, Illumina Human WG-6 v3.0 expression bead chip
	GSE28128 [52]	14	27	CD4+ T cell	GPL8432, Illumina HumanRef-8 WG-DASL V3.0
		LTNPs	HIV + ART		
3 (LA)	GSE18233 [51]	16	29	CD4+ T cell	GPL6884, Illumina Human WG-6 v3.0 expression bead chip
	GSE23879 [2]	12	15	CD4+ T cell	GPL8432, Illumina HumanRef-8 WG-DASL V3.0
	GSE53527 [53]	11	10	CD4+ T cell	GPL10558, Illumina HumanT-12 V4.0 expression bead chip
		HIV-ART	Controls		
4 (HC)	GSE6740 [1]	5	5	CD4+ T cell	GPL96, Affymetrix Human Genome U133A array
	GSE18233 [51]	70	8	CD4+ T cell	GPL6884, Illumina Human WG-6 v3.0 expression bead chip
	GSE9927 [54]	11	9	CD4+ T cell	GPL570, Affymetrix Human Genome U133 plus2.0 array

*GEO* Gene expression omnibus, *GSE* Gene expression series, *No* Number, *HIV-ART* Patients infected with HIV-1 without ART, *HIV + ART* Patients infected with HIV-1 with ART, *LTNPs* Long-term non-progressors, *LC* LTNPs vs. healthy controls, *LH* LTNPs vs. HIV-ART, *LA* LTNPs vs. HIV + ART, *HC* HIV-ART vs. healthy controls

### Meta-analysis

Meta-analyses are necessary to efficiently integrate and validate related datasets produced by independent groups. Meta-analysis step were performed using R version 3.5.0 [12] and Bioconductor 3.6 (http://www.bioconductor.org) with packages, *RankProd* [11] and *limma* [13]. To identify DEGs in samples between LTNPs and others, the datasets of each group were computed using the *RankProd* [11] method, which is a nonparametric approach based on ranks of fold changes (FC). In this approach, FC ratios were computed for all possible pair-wise comparisons for each dataset. The ranks of the ratios were then used to generate a rank product for each gene. Rank products performs well when datasets had low numbers of samples [14]. Permutation tests were then performed to assess the null distributions of the rank products within each dataset. The whole process was repeated multiple times and required powerful computers for the large datasets. We have tested *RankProd* [11] in a workstation (MacPro, 12-core Intel Xeon E5 2.7GHz processors, 64GB memory, macOS Sierra; Apple, Inc., Cupertino California, USA). We used microarray datasets from GEO and these datasets have been pre-processed. The expression values of microarray data were processed using quantile normalization. Data were annotated after converting the gene and probe IDs to the corresponding Entrez IDs and the expression values of same Entrez ID aggregated with mean value. The normalized datasets were then subjected to *limma* [13] to search for DEGs. The intensity values for each probe set were log2 transformed for data integrity.

### Functional enrichment analysis of DEGs

Functional enrichment analysis for DEGs was performed with the Database for Annotation, Visualization and Integrated Discovery (DAVID) [15]. The DAVID is the most common tool to analyze the functional enrichment of genes and identify the significantly enriched GO categories [16]. We also performed the pathway enrichment analysis utilizing the KEGG pathway.

## Results

### Identification of DEGs in the individual microarray data of each group

We calculated DEGs for the individual microarray data in the four groups (Additional file 1). Genes with fold changes (|log_2_FC| > = 1) and *adj. p*-values < 0.05 were considered as differentially expressed. Individual analysis revealed little information correlated with LTNPs and the absence of common DEGs except in the fourth group. It is usually not advisable to directly combine or compare the gene expression values from different gene expression datasets because of their inherent heterogeneity.

### Identification of significant genes using meta-analysis in each group

**Fig. 2:**
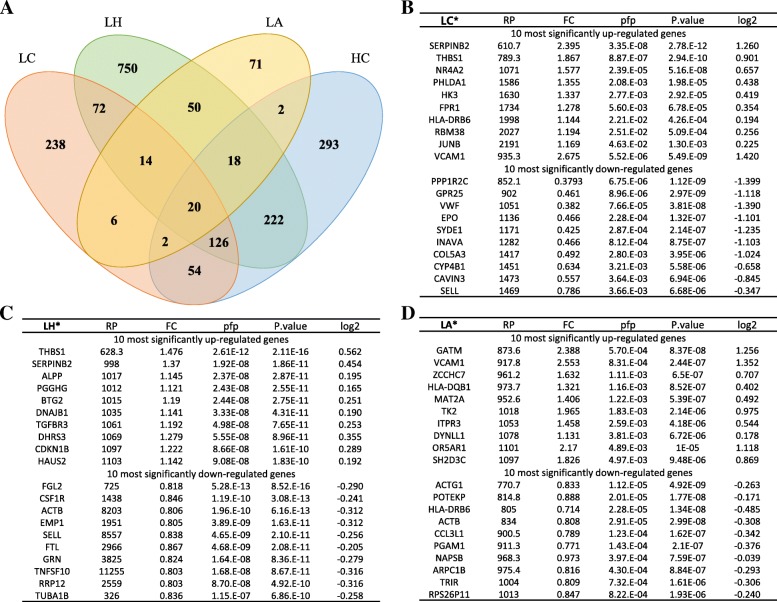
The significant genes in the LC, LH, LA, and HC groups. **a** Venn diagram representing the number of DEGs in LTNPs with the others. **b**, **c** and **d** The 10 most significantly up- and down-regulated genes in each group. Star on group name means that the list of the group does not include genes associated with fourth group

We identified DEGs using meta-analysis in the four groups (Fig. 2a and Additional file 2). The list of DEGs is provided in Additional file 3. To consider only features of LTNPs, we excluded DEGs of the fourth group from DEGs of the three other groups. According to the results of our meta-analysis, 330 genes, 886 genes, and 141 genes were identified to be differentially expressed in the LC group, LH group, and LA group, respectively. Moreover, the 10 most significantly up-regulated and down-regulated genes were identified in each group (Fig. 2b-d). Genes that were identified in the HC group were removed from the list of up- and down-regulated genes.

### Functional enrichment analysis and enriched KEGG pathways for each group

**Fig. 3:**
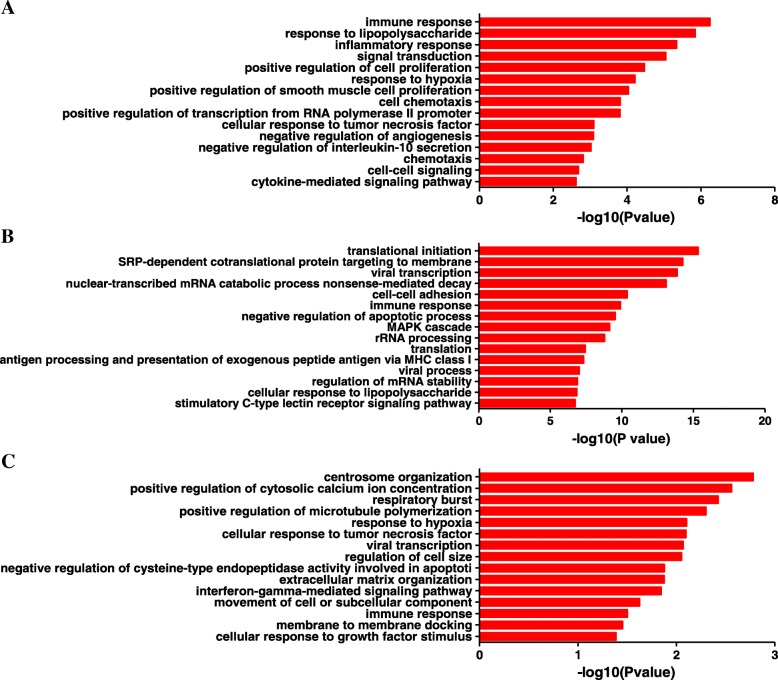
Gene ontology (GO) analysis of DEGs in the three groups **a** The 15 most significantly enriched GO terms for the LC group. **b** The 15 most significantly enriched GO terms for the LH group. **c** The 15 most significantly enriched GO terms for the LA group. *p* < 0.05 was used as the threshold for GO analysis

GO analysis and KEGG pathway analysis for the functional investigation of DEGs were carried out. The significantly top enriched GO terms for DEGs in the LC group included immune response, signal transduction, and positive regulation of cell proliferation (Fig. 3a). In the LH group, translation initiation, viral transcription, and immune response were included (Fig. 3b). Interferon-gamma-mediated signaling pathway and immune response were included in the LA group (Fig. 3c). According to our expectations, immune response was significantly enriched in all three groups.

**Fig. 4:**
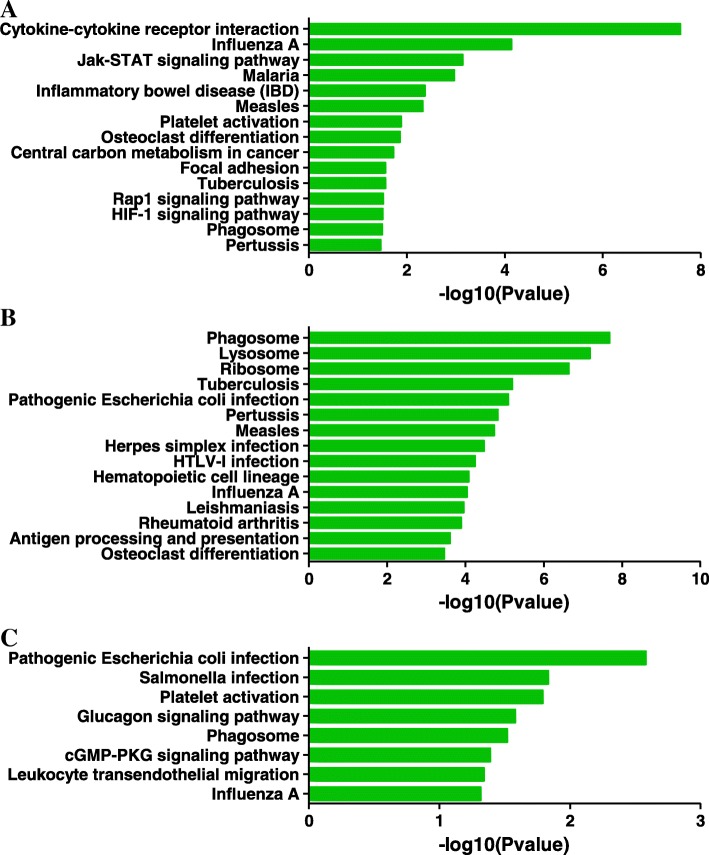
KEGG pathways of DEGs in the three groups. **a** The 15 most enriched pathways in the LC group. **b** The 15 most enriched pathways in the LH group. **c** The 15 most enriched pathways in the LA group

In the pathway analysis, we identified the most significant pathways for DEGs in the three groups. These pathways were as follows: pathways including cytokine-cytokine receptor interaction and Jak-STAT signaling pathway in the LC group, antigen processing and presentation in the LH group, and platelet activation in the LA group (Fig. 4a-c).

### Functional enrichment analysis and enriched KEGG pathways derived from the common DEGs

**Fig. 5:**
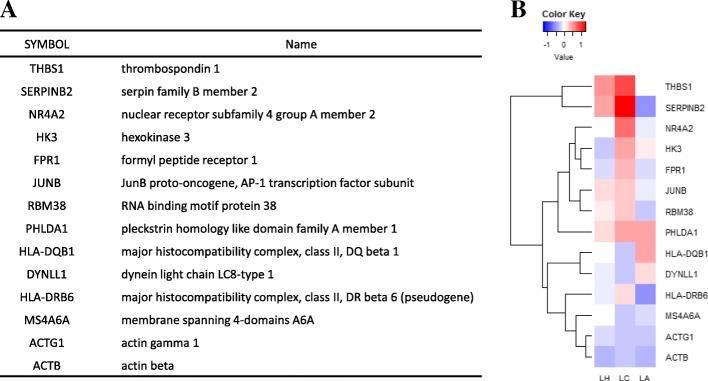
The 14 common genes in the three groups excluding the fourth group. **a** The list of common DEGs. **b** The heatmap of the 14 common DEGs

**Fig. 6:**
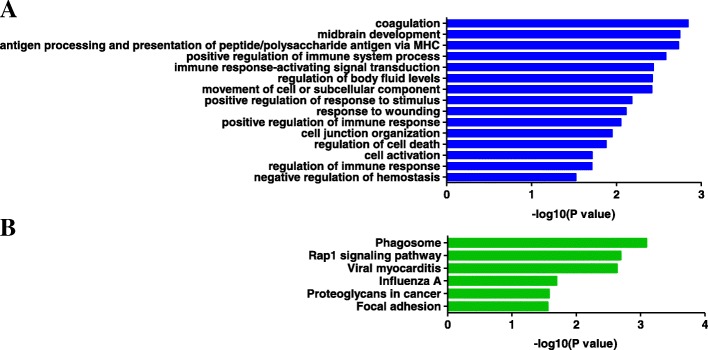
Functional enrichment analysis of the 14 common DEGs. **a** GO-based enrichment analysis of the 14 common DEGs in terms of biological process. **b** KEGG pathways of the 14 common DEGs with *p*-values < 0.05

The significant genes found to be common in all three groups were considered significant genes for LTNPs (Fig. 5a). As a result, 14 genes were identified as common DEGs. Of them, *PHLDA1* was up-regulated and *ACTB* and *ACTG1* were down-regulated in all three groups. However, the up- and down-regulation of the rest of the genes were discordant in the three groups (Fig. 5b). As shown in Fig. 6, antigen processing and presentation, immune response, and immune system process were significantly enriched in the 14 common DEGs related to LTNPs. Moreover, phagosome and Rap1 signaling pathways were identified as the significant pathways for the 14 common DEGs.

## Discussion

LTNPs are a small group of patients who exhibit natural control of HIV-1 viral replication and lack of clinical progression. That is, LTNPs maintain normal counts of CD4+ T cells and controlled viremia without ART for many years. In individuals infected with HIV-1, viral control is a major characteristic of the clinical outcome. Understanding the molecular mechanisms involved in the HIV pathogenesis of LTNPs will provide clues to identify the cause of delayed disease progression in LTNPs. Genome wide association studies (GWAS) were utilized to discover genetic factors and pathways involved in HIV-1 control [17–20]. Tsiara et al. [21] performed meta-analysis of GWAS to investigate the association between interleukin gene polymorphisms and HIV susceptibility. In general, many studies of HIV gene expression have placed emphasis on the comparison in between normal progressors and non-progressors using transcriptional expression profiles [1–5] or meta-analysis [6]. But, in this study, the comparison of gene expression had performed in all the groups, healthy donors, treated HIV-1 patients, non-treated HIV-1 patients and LTNPs.

In our study, we analyzed the gene expression profiles of LTNPs using meta-analysis to understand HIV pathogenic mechanisms. The gene expression profiles of LTNPs were compared with HIV-1 patients, treated HIV-1 patients, and health controls. GO term analysis and KEGG pathway analysis provide understanding of the possible roles of significant genes in the pathogenesis of LTNPs. In all groups, the immune response GO term was significantly enriched. We found that the salmonella infection pathway was significantly enriched in all three groups. Meanwhile, the pathogenic *Escherichia coli* infection pathway was enriched in LH and LA groups. Many researchers have studied the association with bacterial translocation and immune activation of HIV in intestinal microbiome [22, 23]. It was found that microbiota composition is different in between LTNPs and HIV patients [24] and also microbiome dysbiosis is less marked in treated HIV patients and LNTPs than HIV patients [25]. These results suggest that microbiome in LTNPs may have an effect on immune control and HIV disease progression.

We identified the 14 significant common genes. The GO terms of the 14 common genes significantly expressed in LTNPs were associated with immune response, movement of cell, and regulation of cell death. Of them, *THBS1*, *SERPINB2*, *NR4A2*, *DYNLL1*, *FPR1*, *JUNB*, *HLA-DQB1*, *MS4A6A*, *ACTB*, and *ACTG1* have been reported to have interactions with HIV-1 proteins [26–32]. However, little is known about their functions in association with LTNPs. In addition, *HK3*, *RMB38, PHLDA1,* and *HLA-DRB6* had not been previously identified as being related to HIV infection.

HIV-1 gp120 is important for HIV binding to its high-affinity cellular receptor CD4. Thrombospondin 1 (*THBS1*, also known as *TSP-1*), which has biological functions such as in the immune response, activation of MAPK activity and cell adhesion, can directly bind to gp120 in a concentration-dependent fashion. *THBS1* can inhibit HIV infection of PBMCs and transformed T and promonocytic cell lines, presumably through the interaction with the CD36-related TSP-1-binding motif [27, 28]. Consequentially, LTNPs may inhibit HIV entry into host cells and maintain the level of CD4+ T cells through up-regulated expression of *THBS1*. The phylogenetic cluster of envelopes in LTNP-EC patients revealed ineffective binding to CD4, and this modified the signaling activity for actin/tubulin cytoskeletons to result into low fusion and infection capacity [26]. Cytoskeletal components play a major role in various steps of HIV-1 infection. The cytoskeleton contributes to viral assembly and production [29, 30]. Interestingly, *ACTB* and *ACTG1* were found to be down-regulated in LTNPs, and therefore, they may inhibit HIV-1 production, fusion, or entry into host cells. Jayappa et al. [31] found that the knockdown of Dynelin light chain LC8-type 2 (*DYNLL1*) resulted in significantly lower levels of HIV-1 reverse transcription in cells. The expression of *DYNLL1* was down-regulated in the LC and LH groups. Thus, the down-regulated *DYNLL1* in LTNPs may inhibit HIV-1 replication and help maintain a low viral load.

We found that the expression of Pleckstrin homology like domain family A member 1 (*PHLDA1*), also known as T-cell Death-associated gene 51 (*TDAG51*), was significantly up-regulated in all the three groups. It has been implicated in the regulation of cell death and suppression of metastasis in cancers [33–35], and its expression is significantly reduced in human cancers [36, 37]. *PHLDA1* overexpression significantly inhibits the phosphorylation and activation of Akt (also known as protein kinase B) [36, 38]. Akt1 may act to enhance HIV replication through promoting the survival of infected cells [39]. Blocking Akt activation triggered by Nef was found to limit HIV-1 recovery from latently-infected T cells [40], and PI3K/Akt inhibitors were reported to reduce HIV-1 production from infected primary human macrophages [41]. For this reason, the up-regulation of *PHLDA1* in all three groups may inhibit HIV-1 replication through the suppression of the Akt signaling pathway.

RNA binding motif protein (RBM38, also called RNPC1) is known to interact with its target mRNAs and regulate their expression via mRNA stability [42–44]. It is capable of binding and stabilizing the mRNA of p21 [43]. Cyclin-dependent kinase inhibitor 1A (CDKN1A; p21) has been documented for its role in antiretroviral infection [45]. The expression of p21 in human macrophages was induced after HIV-1 infection [46]. In addition, up-regulation of p21 was found in CD4+ T cells from elite controllers [47]. RBM38 may regulate elite controllers through the stability of CDKN1A, a key regulator of HIV infection. Of other DEGs, membrane spanning 4-domains A6A (MS4A6A) has been reported to associate with Alzheimer’s disease [48, 49], and the expression of MS4A6A could be down-regulated by the immunosuppressive domain (gp41) of HIV-1 [50]. Probably, the down-regulation of MS4A6A may reduce new infection of HIV-1 in LTNPs. However, the relationship of these genes with LTNPs should be elucidated in detail.

Our meta-analysis may have some limitations, e.g. limitation of sample size, heterogeneity of microarray platform, and consideration of multifactorial genes interactions. Despite these limitations, our results provide the candidate biomarkers which play a role in the HIV pathogenesis in LTNPs.

## Conclusions

In summary, our meta-analysis identified the 14 significant genes as the biomarkers for LTNPs infected with HIV-1. *THBS1*, *DYNLL1*, *ACTB,* and *ACTG* may control a low viral load. Especially, *PHLDA1* which is unknown to relationship with HIV infection and disease progression may inhibit HIV-1 replication through the suppression of the Akt signaling pathway and delay the disease progression of patients infected with HIV-1. In addition, RBM38 may regulate LTNPs through the stability of CDKN1A and the down-regulation of MS4A6A may modify infection of HIV-1 in LTNPs. Further studies are necessary to elucidate how these genes effects on the mechanisms of delayed disease progression in LTNPs. These results suggest that significant genes identified in a meta-analysis provide clues to the cause of delayed disease progression and give a deeper understanding of HIV pathogenesis in LTNPs.

## Additional files

Additional file 1: **Figure S1.** Venn diagram of the differentially expressed genes (DEGs) identified from each individual microarray analysis. (**A**) The DEGs in LTNPs compared with Healthy Controls; (**B**) The DEGs in LTNPs compared with patients infected HIV-1 without ART; (**C**) The DEGs in LTNPs compared with patients with HIV-1 with ART. (**D**) The DEGs in patients infected HIV-1 compared with Healthy Controls. The up-regulated or down-regulated genes had fold changes (|log_2_FC| > = 1) and *adj. p*-values < 0.05. (PPTX 46 kb)

Additional file 2: **Figure S2.** The estimated pfp (percentage of false prediction) versus the number of identified genes in the LC, LH, LA, and HC groups. The identified genes are marked in red with a cutoff = 0.05. (PPTX 292 kb)

Additional file 3: **Table S1A.** All the significantly down-regulated genes in the LC group. **B**. All the significantly up-regulated genes in the LC group. **C**. All the significantly down-regulated genes in the LH group. **D**. All the significantly up-regulated genes in the LH group. **E**. All the significantly down-regulated genes in the LA group. **F**. All the significantly up-regulated genes in the LA group. **G**. All the significantly down-regulated genes in the HC group. **H**. All the significantly up-regulated genes in the HC group. (XLSX 248 kb)

## Abbreviations

ART: Antiretroviral treatments
DAVID: Database for annotation, visualization and integrated discovery
DEG: Differentially expressed gene
EC: Elite controller
FC: Fold change
GEO: Gene Expression Omnibus
GO: Gene ontology
HC: Patients infected with HIV without ART versus healthy controls
HIV: Human immunodeficiency virus
KEGG: Kyoto Encyclopedia of Genes and Genomes
LA: LTNPs versus patients infected with HIV with ART
LC: LTNPs versus healthy controls
LH: LTNPs versus patients infected with HIV without ART
LTNP: Long-term non-progressor
PBMC: Peripheral blood mononuclear cell
