# Supplementary material for: Meta-analysis of gene expression profiles in long-term non-progressors infected with HIV-1
Source: BMC Med Genomics. 2019 Jan 9;12:3. doi: 10.1186/s12920-018-0443-x (PMC6325803; doi:10.1186/s12920-018-0443-x)
Supplement: Supplementary file 1 — Figure S1. Venn diagram of the differentially expressed genes (DEGs) identified from each individual microarray analysis. (A) The DEGs in LTNPs compared with Healthy Controls; (B) The DEGs in LTNPs compared with patients infected HIV-1 without ART; (C) The DEGs in LTNPs compared with patients with HIV-1 with ART. (D) The DEGs in patients infected HIV-1 compared with Healthy Controls. The up-regulated or down-regulated genes had fold changes (|log2FC| > = 1) and adj. p-values < 0.05. (PPTX 46 kb) [file 12920_2018_443_MOESM1_ESM.pptx]

## Slide 1
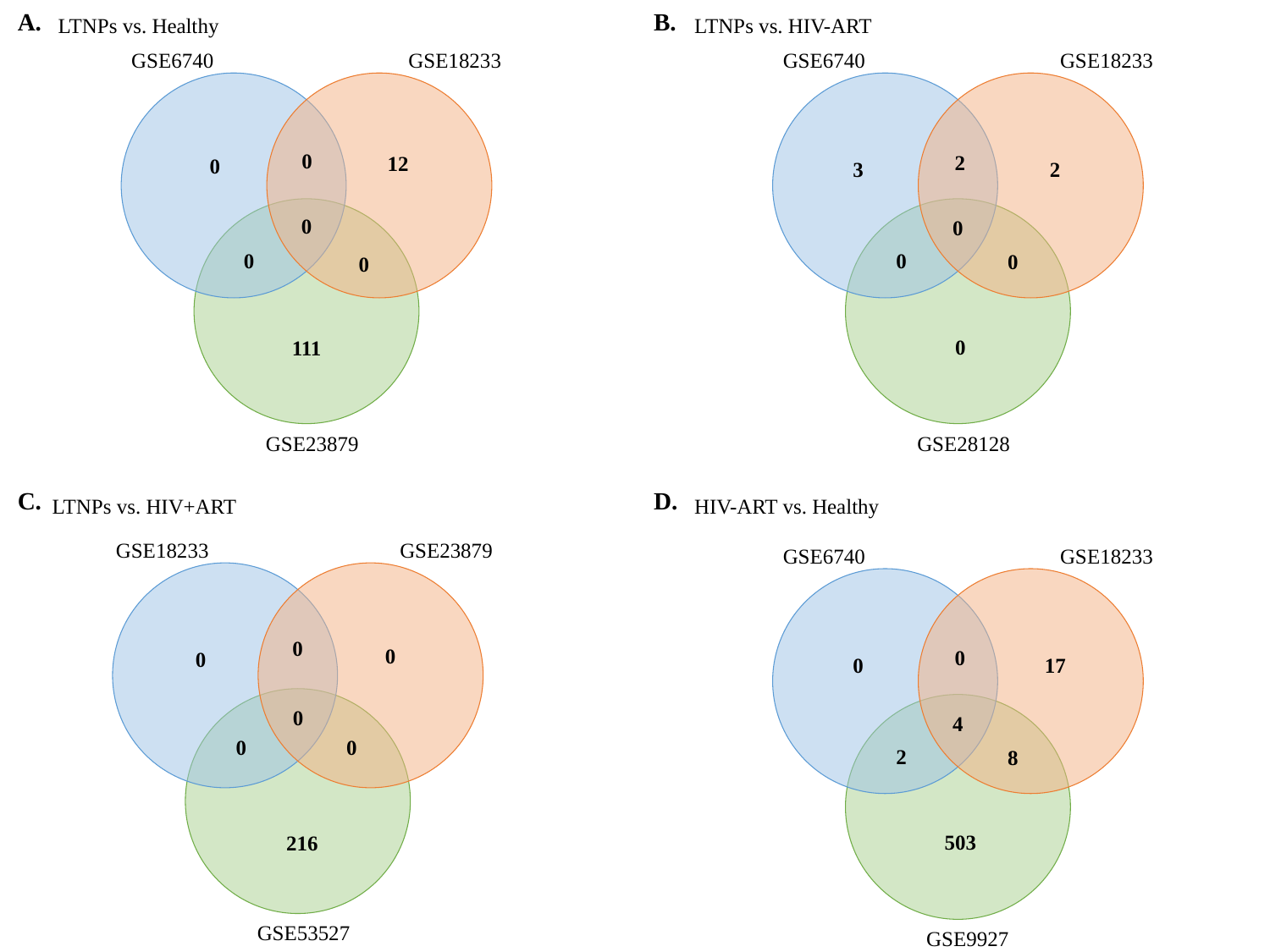

A.
B.
LTNPs vs. HIV-ART
LTNPs vs. Healthy
GSE18233
GSE6740
GSE23879
0
12
0
0
0
0
111
GSE18233
GSE6740
GSE28128
2
3
2
0
0
0
0
C.
D.
HIV-ART vs. Healthy
LTNPs vs. HIV+ART
GSE23879
GSE18233
GSE53527
0
0
0
0
0
0
216
GSE18233
GSE6740
GSE9927
0
0
17
4
2
8
503
