# Supplementary figures and images for: Meta-analysis of gene expression profiles in long-term non-progressors infected with HIV-1
Source: BMC Med Genomics. 2019 Jan 9;12:3. doi: 10.1186/s12920-018-0443-x (PMC6325803; doi:10.1186/s12920-018-0443-x)

## Slide 1
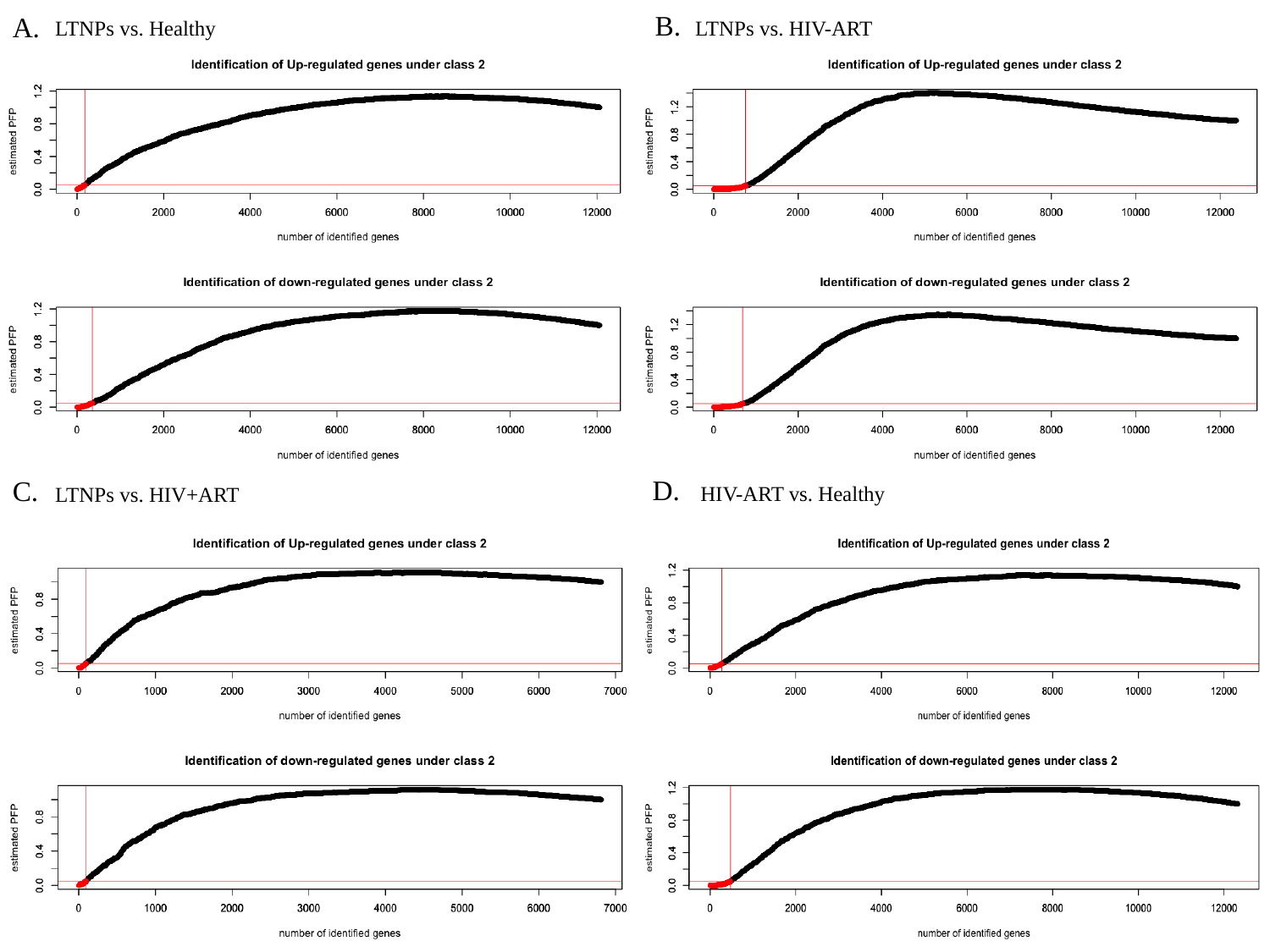

B.
A.
LTNPs vs. Healthy
LTNPs vs. HIV-ART
D.
C.
HIV-ART vs. Healthy
LTNPs vs. HIV+ART

Supplement: Supplementary file 2 — Figure S2. The estimated pfp (percentage of false prediction) versus the number of identified genes in the LC, LH, LA, and HC groups. The identified genes are marked in red with a cutoff = 0.05. (PPTX 292 kb) [file 12920_2018_443_MOESM2_ESM.pptx]
